# Socioeconomic risks of food insecurity during the Covid-19 pandemic in the UK: findings from the Understanding Society Covid Survey

**DOI:** 10.1186/s12889-022-12964-w

**Published:** 2022-03-26

**Authors:** Heather Brown, Susanna Mills, Viviana Albani

**Affiliations:** 1 Newcastle University, Population Health Sciences Institute and Fuse -Centre for Translational Research in Public Health, Newcastle upon Tyne, England, UK; 2 Present Address: Finnish Institute of Health and Welfare, Mannerheimintie 166, 00300 Helsinki, Finland

**Keywords:** Food insecurity, Covid-19, UK, Financial vulnerability

## Abstract

**Background:**

We estimated socioeconomic factors associated with food insecurity during the first year of the Covid pandemic in the UK and explored potential mechanisms explaining these associations.

**Methods:**

Data were from the April, July, and September 2020 waves of the UK Understanding Society Covid Survey. Food insecurity was measured as ‘not having access to healthy and nutritious food’ and ‘reporting being hungry but not eating’. Logistic regression estimated the relationship between socioeconomic factors and food insecurity. A decomposition approach explored if financial vulnerability and having Covid-19 explained associations between socioeconomics factors and food insecurity.

**Results:**

Single parents and young people aged 16–30 years had a higher odds of reporting both measures of food insecurity. Financial insecurity explained 5% to 25% of the likelihood of reporting being food insecure for young people and single parents depending on the food insecurity measure used. Experiencing Covid-19 symptoms explained less than 5% of the likelihood of being food insecure for single parents but approximately 30% of not having access to healthy and nutritious food for young people.

**Conclusion:**

Policies providing additional financial support may help to reduce the impact of Covid-19 on food insecurity in the UK.

**Supplementary Information:**

The online version contains supplementary material available at 10.1186/s12889-022-12964-w.

## Introduction

The Covid-19 pandemic associated with SARS-CoV-2 virus has changed the way we live globally. The pandemic has led to governments across the world implementing social distancing measures and restrictions on movement and work, generally referred to as “lockdowns”, at various stages during the pandemic in an attempt to contain viral spread [1]. The associated impact of putting the brakes on economic activity has had a profound effect on economic growth. Global economic growth in 2020 was 4.9% lower than in 2019 and it is projected that global Gross Domestic Product (GDP) in 2021 will be 6.5% lower than pre-Covid-19 projections made in January 2020 [2]. These huge macro-level economic changes in the role of the state in economic decision making such as which businesses can operate associated with containment measures to reduce the spread of the virus as well as risks from the virus have had significant impacts on individual behaviour and both health and social outcomes.

In the UK, the fear and uncertainty associated with Covid-19 led to a change in consumer spending in the first three weeks of March 2020 leading up to the first national lockdown [3–5]. There were large increases in consumer spending on food staples such as canned goods, dried pasta, flour, and soup [5]. As a result of unanticipated increase in demand in late February and early March 2020, by the start of the first UK national lockdown, 40% of people had difficulty accessing the basic food they required at supermarkets [6]. Thus, the number of people reporting being food insecure quadrupled in March 2020 in the UK [6]. Here, the term food insecurity relates to both physical and economic access to food. For a person to be considered food secure they need to have sufficient quantities of food available on a consistent basis, adequate income or resources available to access appropriate food, and adequate food must be available at all times with access to and availability of food not curtailed by acute or recurring emergencies [7]. This definition excludes the use of food aid such as food banks. People may use food banks because they are food insecure or at risk of food insecurity. However, not everyone who is unable to access healthy and nutritious food or has insufficient quantities of food may utilise food aid, hence usage of aid is likely to underrepresent need.

In 2015, all United Nation (UN) member states signed up to achieving the 17 UN Sustainable Development Goals (UNSDGs) by 2030 [8]. UNSDG 2 is zero hunger; aiming to eliminate food insecurity by 2030. Before the pandemic, in 2016, it was estimated that approximately 8.4 million households in the UK were food insecure [9]. In 2019, the Government’s voluntary review on progress towards achieving SDG 2 identified that the UK was making insufficient progress to achieve zero hunger by 2030. Increases in food insecurity are likely to be a driving factor in the dramatic rise in the demand for food aid such as food banks in the UK. There were an estimated 65 foodbanks in the UK in 2011 which grew to over 1,200 in 2019 [10]. This demonstrates that even before the arrival Covid-19, the UK was significantly lagging behind in its aim to reduce food insecurity.

There is currently a lack of UK quantitative data available on the determinants of food insecurity in the UK. However, a report commissioned by the foodbank charity the Trussell Trust [10], found the following risk factors for being food insecure in the UK before the Covid-19 pandemic: low income, single parent household, being of working age, living alone, renting one’s home, being unemployed, and living in a household affected by poor health. Poverty is strongly associated with food insecurity, however not all households that are food insecure are in poverty and not all households in poverty are food insecure [11].

In the first year of the pandemic in 2020, the UK Government implemented a range of support measures in an attempt to mitigate the associated economic consequences. These support measures—which have been revised as the pandemic has progressed—included a self-employed income support scheme, a job retention scheme – through which the Government helped to pay part of the salary of ‘furloughed’ employees—a freezing of business rates, a grant scheme for small businesses, and business interruption loans [12]. From the 6^th^ April 2020, two of the most important state welfare benefits -tax credits and universal credit- increased by £20 a week for one year to help low-income households [13]. For children eligible for free school meals, the government created a £15 weekly food voucher scheme facilitated through schools to be given to families [14]. This scheme was extended from the school term to include the summer holidays in 2020 after a successful awareness-raising campaign led by the professional footballer Marcus Rashford [15]. These measures are likely to prevent some households falling into poverty and financial strain, with potential avoidance of food insecurity.

However, there are multiple gaps in these economic safety nets as new employees and the self-employed, those who pay themselves in dividends, those earning a mix of salary and self-employed income, freelancers, and sole traders are not eligible for support [16]. The impact of this is demonstrated by evidence indicating that only a very small minority of people actually self-isolate or quarantine appropriately in line with UK national Covid requirements; this contrasts with very high reported intentions to adhere to guidance [17]. Houston [18] found that those who were unemployed pre-Covid were more likely to remain unemployed and not find a job at the start of the pandemic. Thus, these groups are in a weakened position to deal with the associated economic fallout. There is evidence showing that women, single parents, young people (aged 16–30 years), and ethnic minorities have borne the brunt of the economic impact of the pandemic [19]. This suggests that the Covid pandemic has widened existing inequalities and may be expected to further exacerbate inequalities in future, with associated negative health and social implications.

The policy measures put in place to mitigate the economic impact of Covid-19, such as the furlough scheme [12] have predominantly supported those who were in employment before the pandemic. The protective effects of the furlough scheme on employment are demonstrated by the fact that employment in sectors unable to operate under lockdown explains only 5.7% of the increase in variation in unemployment during the Covid-19 pandemic between areas of the UK [18].

We focus on access to food and reporting being hungry as our measures of food insecurity. The Covid-19 pandemic increased supply side issues related to the availability of food because of both changes in consumer behaviour (hoarding/panic buying) [5, 6] as well as containment and quarantine measures which may have impacted on people’s access to food. Being hungry and not being able to eat may have increased for those who were financially constrained and then fell through the economic social safety net provisions in place which is why we focus on this measure. Given the nature of government support available during the Covid-19 pandemic we hypothesise that people who are more economically and medically vulnerable to Covid-19 are more likely to report being food insecure. It is likely that those who had higher likelihood of being food insecure before the pandemic such as those on low income, single parents, young people, people living in a household with someone in poor health [10] will be at increased likelihood of suffering from food insecurity during the Covid-19 pandemic because of risk factors associated with increased vulnerability to both the economic and health consequences of the virus. We hypothesise that including a range of different socioeconomic factors related to the social determinants of health such as educational attainment, household composition, current employment status, gender, age, marital status, housing tenure type, and reporting a limiting long-term condition will be associated differentially with the two measures of food insecurity, since these measures are capturing different aspects of what it means to be food insecure. For example, access to food may affect people across the socioeconomic spectrum and could be related to other factors such as rurality whereas being hungry and not eating is more likely to be associated with characteristics related to economic vulnerability. Having covid-19 symptoms may mean people need to isolate which may impact on their ability to access food. If people are not compensated for when they isolate this could impact on their ability to afford food.

The aim of this paper is to explore which economic and socioeconomic factors are associated with experiencing the two measures of food insecurity 1) access to healthy and nutritious food and 2) being hungry but not able to eat. Next, for those characteristics associated with increased likelihood of food insecurity we explore to what extent financial vulnerability and having Covid-19 symptoms explain this increased risk. We focus on symptoms rather than diagnosis because availability of Covid-19 tests changed over the pandemic period and some people may not have been able to access a test. Thus, to include all survey respondents who may have been affected by Covid we decided to include the broadest possible definition. To achieve these aims we use a national longitudinal dataset from the UK, the Understanding Society Covid Survey.

## Methods

### Data

We use data from waves 1, 4, and 5 (April, July, and September 2020) of the Understanding Society Covid Survey from the UK [20]. These are the three waves in which questions on food insecurity were asked of participants. The Covid survey is a sub- survey of approximately 17,000 households who participated in the main Understanding Society Survey, which is an annual household longitudinal survey of 40,000 households [21]. The Covid survey ran monthly from April 2020 until March 2021 with an additional antibody testing kit in April 2021 and a closing survey in summer 2021. The aim of the survey is to understand the impact of the pandemic on individuals, families, and communities. The University of Essex Ethics Committee for the COVID-19 web and telephone surveys (ETH1920-1271) granted ethical approval for the study. All methods were carried out in accordance with relevant guidelines and regulations.

The analysis is limited to respondents who respond to all study questions of interest in at least one wave of data collection. This gives us 9,501 observations for our first measure of food insecurity and 9499 observations for our second measure of food insecurity.

### Measure of food insecurity

There is no single widely accepted definition of food insecurity [22]. To proxy for food insecurity, we focus on quantity and access to sufficient food based upon the Food and Agricultural Organisation of the United Nations definition of food insecurity [23] whereby a person is food insecure if they report either of the following:

1. Being hungry and not able to eat
2. Inability to access sufficient and nutritious food because of lack of money or other resources

We record these as binary variables which are equal to one if the respondent reports yes to either or both of the two questions above and equal to zero if the respondent reports no. A person might not necessarily report both measures of food insecurity. These measures of food insecurity have not been validated to date in this population. However, they can provide useful insights on factors associated with the two elements of food insecurity, namely access and affordability, which has important implications for public health.

### Socioeconomic factors influencing the likelihood of being food insecure

The explanatory variables used in the analysis are drawn from the wider literature identifying the determinants of food insecurity in high income countries [24–26] and the social determinants of health [27]. The variables we include in our models are log of equivalised household income, a binary variable which equals one if an individual owns or has a mortgage on their house and is equal to zero if they rent, binary variables for educational attainment, a binary variable for long term sick/disabled, a binary variable for being a young person between 16–30 years of age, and a binary variable for being a single parent. We control for additional demographic factors through a binary variable for being female, and binary variables for having children between ages 0–2, 3–4, 5–11, and 12–15 years.

### Financial vulnerability and Covid-19 symptoms

We explore two pathways to explain the association between the identified socioeconomic factors and food insecurity. To control for financial vulnerability or the likelihood of being negatively financially impacted by containment measures to reduce the spread of the virus we include a variable on the reduction in working hours and being in debt (defined as being behind on bills). To control for Covid-19 symptoms we include a binary variable of having Covid-19 symptoms.

### Statistical analysis

To estimate the relationship between socioeconomic factors and food insecurity, we start by estimating logistic regressions for the two measures of food insecurity. Standard errors are clustered to control for the fact that we have observations on some individuals at more than one time point. The analysis is pooled across all three waves of data.

Next, for the characteristics that we identify as increasing the likelihood of being at risk for being food insecure we explore to what extent financial vulnerability and having Covid-19 symptoms explain this risk. To do this we employ a decomposition approach following Kohler, Karlson, and Holm [28]. This approach allows us to determine if there is an indirect or moderating effect of having Covid-19 symptoms and economic vulnerability on the relationship between food insecurity and sociodemographic factors. The model decomposes how much of the observed correlation between the key explanatory variable is explained by having Covid-19 symptoms and economic vulnerability and how much stems from other individual characteristics. A logit model is used to extract the residuals to be able to separate out the direct and indirect effects explaining the observed association.

We pooled the data so that the results would be consistent across our two different estimation approaches as the decomposition approach that we employ cannot account for changes over time.

The STROBE checklist for observational studies is followed and can be found in Additional file 1: Appendix A.

## Results

The descriptive statistics for the dataset covering all survey waves used in this study are presented in Table 1. Slightly over half of the sample was female (58%, *n* = 5510). Approximately 15% (*n* = 1425) of the sample was aged between 16–30 years, 5% (*n *= 475) of the sample were single parents. Approximately 82% (*n* = 7790) of the sample owned or had a mortgage on their home. Approximately 13% (*n* = 1235) of the sample reported someone in the household being unable to eat healthy or nutritious food but only 2% (*n* = 190) reported being hungry and not eating. Approximately 10% (*n* = 950) of the sample were furloughed, 6% (*n* = 570) reported having Covid-19 symptoms, and 8% (*n* = 760) were newly unemployed. Comparing this with the main Understanding Society Survey [29] and other national surveys [30], this sample is more affluent than the general UK population.

**Table 1:** Descriptive statistics

Variables	Mean/Standard Deviation/Observations (n*T)
Pre-pandemic household characteristics	
female	0.58 (0.49) 9,501
A-level (some higher education)	0.22 (0.41) 9,501
GSCE (basic qualifications)	0.19 (0.40) 9,501
no qualifications	0.05 (0.21) 9,501
Annual equivalised household income	21,655.32 (14,247.28) 9,501
Disabled/Long Term Sick	0.03 (0.18) 9,501
Has children in house between 0–2 years old	0.08 (0.27) 9,501
Has children in house between 3–4 years old	0.06 (0.24) 9,501
Has children in house between 5–11 years old	0.17 (0.38) 9,501
Has children in house between 12–15 years old	0.13 (0.34) 9,501
Own outright or mortgage on property	0.82 (0.38) 9,501
Young Person	0.15 (0.36) 9,501
Single Parent	0.05 (0.22) 9,501
Pandemic related variables	
Any person in the household unable to eat healthy and nutritious food	0.13 (0.34) 9,501
Hungry but did not eat	0.02 (0.15) 9,499
Newly Unemployed	0.08 (0.10) 9,501
Late with bills	0.05 (0.22) 9,501
Furloughed	0.11 (0.31) 9,501
Covid-19 symptoms	0.06 (0.23) 9,501

Table 2 shows the results from the logistic regressions identifying the demographic and economic factors that are associated with an increased risk of food insecurity during the Covid-19 pandemic. Column 1 shows the results for access to healthy and nutritious food and column 2 shows the results for being hungry but not able to eat. Being a single parent is associated with 2.85 higher times of not having access to healthy and nutritious food and 3.39 higher times reporting being hungry but not able to eat. Being a young person (aged 16–30 years) compared to an older adult is associated with 4.12 times higher likelihood of reporting being hungry but not able to eat. Having no qualifications compared to any type of educational qualifications is associated with 6.96 higher times of reporting being hungry but not able to eat. Furloughed workers were associated with 2.73 higher times of reporting being hungry but not able to eat whereas there was a significant negative association with reporting having access to healthy and nutritious food. Individuals with more economic resources (owning or having a mortgage on their home and higher household income) had a significantly negative association with reporting being hungry but not able to eat. Those with children aged 12–15 years compared to those with children of other ages or not having children at all was significantly and negatively associated with reporting not having access to healthy and nutritious food.

**Table 2:** Logistic regressions of the risk of reporting food insecurity during the Covid-19 pandemic

	(1)	(2)
Variables	Access	Hungry
Female	1.02 (0.08)	1.02 (0.24)
Disabled/long term sick	1.70 (1.31)	8.02 (14.18)
Furloughed	0.34*** (0.07)	2.73*** (0.79)
Single Parent	2.85*** (0.44)	3.39*** (1.54)
Young person	1.13 (0.12)	4.12*** (1.16)
Has kids 0–4	0.88 (0.13)	0.93 (0.37)
Has kids 5–11	1.13 (0.11)	1.19 (0.31)
Has kids 12–15	0.76** (0.09)	1.18 (0.35)
Owns/mortgage house	0.87 (0.09)	0.32*** (0.08)
Log of equivalised household income	0.91 (0.06)	0.56*** (0.10)
No qualifications	0.99 (0.37)	6.94*** (4.69)
Basic (GCSE)	1.08 (0.12)	1.56 (0.47)
Some higher qualifications (A-level)	0.91 (0.10)	1.14 (0.32)
Constant	0.24** (0.17)	0.45 (0.77)
Observations	9,501	9,499
Number of individuals	6,302	6,300

*Notes*: Odds ratios are shown. Standard errors in parentheses. *** *p* < 0.01, ** *p* < 0.05, * *p* < 0.1

**Fig. 1:**
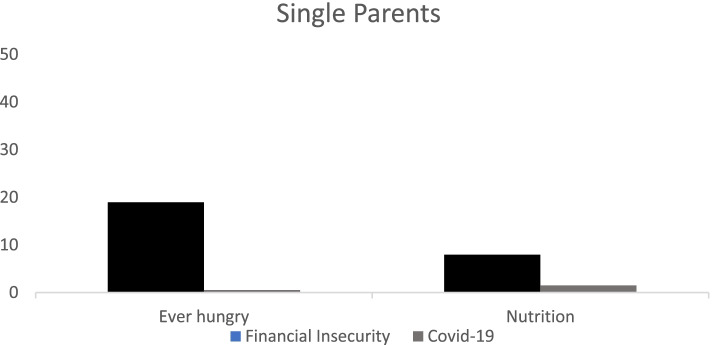
Percent Contribution of financial insecurity and having Covid-19 symptoms on reporting being hungry and not being able to eat and not having access to healthy and nutritious food for single parents

**Fig. 2:**
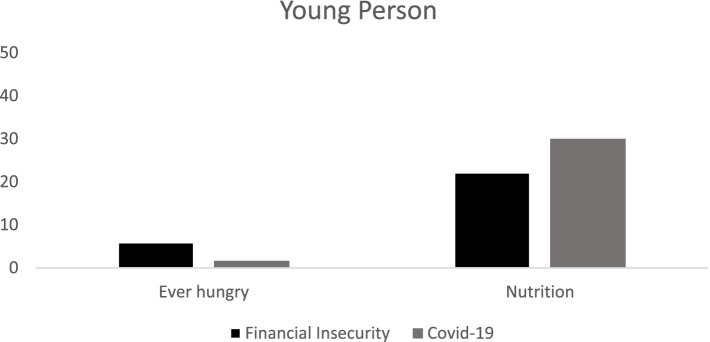
Percent Contribution of financial insecurity and having Covid-19 symptoms on reporting being hungry and not being able to eat and not having access to healthy and nutritious food for young people (aged 16–30 years)

We then explored to what extent the association we found for the two measures of food insecurity for single parents (Fig. 1) and for young people (Fig. 2) is explained by financial vulnerability and having Covid-19 symptoms employing the Kohler et al. [28] decomposition approach for non-linear variables. For single parents in Fig. 1, financial vulnerability (as defined by a reduction in working hours and being behind on bills after controlling for employment status), explained approximately 20% of the likelihood of reporting ever going hungry and 5% of lacking access to healthy and nutritious food. For single parents, having Covid-19 symptoms explained less than 5% of the likelihood of reporting the two measures of food insecurity. For young people in Fig. 2, financial vulnerability explained approximately 5% of the likelihood of reporting going hungry and 25% of the likelihood of reporting not having access to healthy and nutritious food. For this group, having Covid-19 symptoms explained less than 5% of the likelihood of going hungry but approximately 30% of the likelihood of reporting not having access to healthy and nutritious food.

## Discussion

### Main finding

In this paper, we explore socioeconomic factors associated with the two measures of food insecurity during the Covid-19 pandemic in the UK. We find that single parents compared to partnered parents or childless people and those who are furloughed compared to those who continue to work are significantly associated with reporting both measures of food insecurity. It is important to note that being furloughed was negatively associated with difficulty in accessing food but positively associated with reporting being hungry but not able to eat. Young people compared to those who are aged over 30 years and those who have no qualifications compared to those with some formal educational qualifications are significantly associated with reporting being hungry but no association with access to food was observed. We further explore to what extent financial vulnerability experienced during the pandemic can explained the increased risk of food insecurity experienced by young people and single parents. We find that financial vulnerability explains between 5 to 25% of the likelihood of reporting being food insecure for young people and single parents. These percentages are dependent upon the measure of food insecurity. Next, we estimated the impact of having Covid-19 symptoms on the likelihood of being food insecure for young people and single parents. Having Covid-19 symptoms explains less than 5% of the likelihood of being food insecure for single parents but explains approximately 30% of not having access to healthy and nutritious food for young people.

### What is already known on the topic

Growing levels of food insecurity pre-pandemic have been associated with eating cheaper and less healthy food [31]. There is a global evidence suggesting that there is an association between childhood food insecurity and obesity [32, 33] and adult obesity and food insecurity [34, 35]. If more people are pushed into food insecurity, this is likely to impact on the UK government’s ability to reach their target of reducing adult obesity and halving childhood obesity by 2030 [36] in addition to undermining its commitment to the UN SDG2 goal.

There have been two studies which are particularly relevant for analysis related to financial vulnerability and food insecurity during the first year of the Covid-19 pandemic. For the UK, Joyce and Xu [19] found that the economic impacts of the Covid-19 pandemic are likely to have a larger effect on young people and women, who typically tend to work in sectors impacted by lockdowns such as hospitality and retail. Low earners and those without other working household members (e.g. single parents) are also more likely to experience a negative impact on their finances. This is consistent with our results that showed that vulnerable groups were more likely to report one or both measures of food insecurity and the financial vulnerability may be one mechanism explaining this observed association. Milovanska-Farrington [37] has shown that in the USA, unemployment was associated with increased risk for food insecurity and was there was a higher risk for those who were already unemployed at the start of the pandemic. In our analysis we do not investigate those who are unemployed but explore those who are on the furlough scheme (not working but receiving a proportion of their former salary). We found being on the furlough scheme to be positively associated with reporting going hungry but interestingly was negatively associated with difficulty in accessing food which is different to that found in Milovanska-Farrington [37]. This may be because people on the furlough scheme thought the pandemic and associated containment measure was a temporary situation so were willing to go hungry for a short period of time and would be returning to work in the short to medium term. Whereas in the Milovanska-Farrington study the sample were those who were unemployed so these people may have been less confident about their short to medium term finances.

### What this study adds

We found that in comparison with those who continued working those who were furloughed during the Covid-19 pandemic in the UK were more likely to report being hungry but not able to eat compared to those who were still working. Furloughed people were less likely to have difficulties in access to healthy and nutritious food compared to those who were still working. The furlough scheme has helped to keep unemployment lower than was otherwise forecasted by Office of Budget Responsibility [38]. Thus, successfully keeping some families out of food insecurity over the past 18 months. The furlough scheme and other government support provided such as the £20 uptick in Universal Credit has done much to help mitigate the negative economic consequences of the Covid-19 pandemic. Hence if a person is eligible for any government support or redundancy payment, then reducing working hours or becoming unemployed may not necessarily negatively impact on finances in the short term. However, these schemes will close by the end of 2021, so it is important to understand which population groups are economically vulnerable and how this vulnerability relates to food insecurity.

In terms of needing to self-isolate due to Covid-19 symptoms as a factor contributing to food insecurity; the UK has the lowest level of statutory sick pay in the Organisation for Economic Cooperation and Development (OECD) [39]. There is also little practical and additional financial support available to those who need to self-isolate, particularly for people who are not already eligible for government benefits [40, 41]. This may explain our finding that having Covid symptoms explained 30% of the likelihood of not having access to healthy and nutritious food for young people; since when self-isolating they may not have been able to purchase the food they required.

In addition, logistical issues impacting on the supply of food in the UK, in Autumn 2021, has reduced donations of food from both supermarkets and the public. This will limit food banks and other charities’ ability to support those who do require food aid. This highlights the pressing need to ensure that more people do not end up in a position where they require food aid.

The lack of UK quantitative data on food insecurity, means that to date, this is an issue that is not well understood in the UK. The Understanding Covid Survey provides a novel opportunity to explore risks of food insecurity during the pandemic. We are able to investigate two dimensions of food insecurity: access and insufficient resources [23]. People may have difficulty accessing food but do not lack the resources to purchase food if available and vice versa. Thus, it is important to understand what socioeconomic characteristics are associated with each dimension. The policy responses to each dimension of food insecurity are also likely to be different. Thus, it is important to understand who is at risk for both dimensions of food insecurity and how this could be mitigated. However, there are some broader difficulties with putting this into a wider context given the existing unknowns about food insecurity pre-pandemic.

Our findings can be used in the future to expand the provision of economic and social safety nets, to decrease the likelihood that those who are most at risk become food insecure. In turn, this will help to put the UK back on track to reach UN SDG2 of eliminating hunger, as well as mitigating the negative wider social and health consequences of the Covid-19 pandemic.

### Limitation

Due to the complexity of measuring food insecurity, it is possible that the proxies used in our analyses may not have captured all food insecure households. The measures of food insecurity we employ have not been validated for use in this population. However, the Understanding Society Survey questions have been widely applied in international research and practice literature, thereby increasing the potential comparability of our findings [30]. The survey data overrepresents women and those who are more affluent which may reduce the generalisability of our findings. We are also unable to look at changes over time in our analysis. Future research should explore how food insecurity, and the factors contributing to food insecurity, change over time, as the economic consequences of the Covid-19 pandemic unfold.

## Conclusion

Our research finds that single parents are more likely to report both measures of food insecurity employed in this study. Young people and those on the furlough scheme are more likely to report being hungry but not able to eat. The relationship between financial vulnerability and experiencing Covid-19 symptoms vary depending on the measure of food insecurity studied, and are different for single parents and young people. Young people and single parents experiencing food insecurity should be engaged to help shape future interventions to reduce food insecurity in these vulnerable groups.

## Supplementary Information

**Additional file 1.** Appendix A.

## Data Availability

The data that support the findings of this study are openly available in [the UK data archive] at https://10.5255/UKDA-SN-8644–10 reference number [SN-8644–10].

## References

[1] Devereux S, Béné C, Hoddinott J (2020). Conceptualising COVID-19’s impacts on household food security. Food Security.

[2] International Monetary Fund 2021. World economic outlook report. Available from: https://www.imf.org/en/Publications/WEO. Accessed Apr 2021.

[3] Bachas N, Ganong P, Noel PJ, Vavra JS, Wong A, Farrell D, Greig FE. Initial impacts of the pandemic on consumer behavior: evidence from linked income, spending, and savings data. Cambridge: National Bureau of Economic Research; 2020.

[4] Baker SR, Farrokhnia RA, Meyer S, Pagel M, Yannelis C (2020). How does household spending respond to an epidemic? Consumption during the 2020 COVID-19 pandemic. Rev Asset Pricing Stud.

[5] O’Connell M, De Paula Á, Smith K. Preparing for a pandemic: spending dynamics and panic buying during the COVID-19 first wave. Fisc Stud. 2021;42(2):249–64. 10.1111/1475-5890.12271 PMC8209877 34176997

[6] Loopstra R. Vulnerability to food insecurity since the COVID-19 lockdown. London: The Food Foundation. 2020 Apr 14. Available from: https://foodfoundation.org.uk/publication/vulnerability-to-food-insecurity-since-the-covid-19-lockdown/. Accessed Oct 2020.

[7] World Health Organisation 2021. Nutrition food systems. Available from: http://www.emro.who.int/nutrition/food-security/. Accessed Apr 2021.

[8] United Nations . Sustainable development goals. Available from: https://www.un.org/sustainabledevelopment/development-agenda/. Accessed May 2021.

[9] Taylor A, Loopstra R. Too poor to eat: food insecurity in the UK. London: The Food Foundation; 2016.

[10] Sosenko F, Littlewood M, Bramley G, Fitzpatrick S, Blenkinsopp J, Wood J. State of hunger: a study of poverty and food insecurity in the UK. Salisbury: Trussell Trust; 2019.

[11] Wight V, Kaushal N, Waldfogel J, Garfinkel I (2014). Understanding the link between poverty and food insecurity among children: does the definition of poverty matter?. J Child Poverty.

[12] Gov.uk. Chancellor's statement on coronavirus (COVID-19): 26 March 2020. Available from: https://www.gov.uk/government/speeches/chancellor-outlines-new-coronavirus-support-measures-for-the-self-employed. Accessed Nov 2020.

[13] Reddy Siddiqui. Available from: https://www.reddysiddiqui.com/coronavirus/covid-19-increased-support-available-through-tax-credits-and-universal-credits. Accessed Nov 2020.

[14] Gov.uk. Guidance Covid Summer Food Fund 2020. Available from: https://www.gov.uk/guidance/covid-summer-food-fund. Accessed Nov 2020.

[15] Save the children. Building on Marcus Rashford’s campaign to offer a lifeline for children. Available from: https://www.savethechildren.org.uk/blogs/2020/marcus-rashford-free-school-meals-campaign-lifeline. Accessed Nov 2020.

[16] Greater London Authority. Gaps in the self-employment income support scheme. Available from: https://www.london.gov.uk/questions/2020/3902. Accessed May 2021.

[17] Smith LE, Amlȏt R, Lambert H, Oliver I, Robin C, Yardley L, Rubin GJ (2020). Factors associated with adherence to self-isolation and lockdown measures in the UK: a cross-sectional survey. Public Health.

[18] Houston D (2020). Local resistance to rising unemployment in the context of the COVID-19 mitigation policies across Great Britain. Reg Sci Policy Pract.

[19] Joyce R, Xu X. Sector shutdowns during the coronavirus crisis: which workers are most exposed. Institute for Fiscal Studies Briefing Note BN278. 2020 Apr;6. Available from: https://www.ifs.org.uk/publications/14791/. Accessed Apr 2021.

[20] University of Essex, Institute for Social and Economic Research. (2020). Understanding society: COVID-19 study, 2020. [data collection]. 4th Edition. UK Data Service. SN: 8644, 10.5255/UKDA-SN-8644-4.

[21] University of Essex, Institute for Social and Economic Research, NatCen Social Research, Kantar Public. (2019). Understanding Society: Waves 1-9, 2009-2018 and Harmonised BHPS: Waves 1-18, 1991-2009. [data collection]. 12th Edition. UK Data Service. SN: 6614, 10.5255/UKDA-SN-6614-13.

[22] Coates J. Reaching for the stars?: Universal measures of household food security. In Annals of Nutrition and Metabolism 2009 Jan 1 (Vol. 55, pp. 69–69). Allschwilerstrasse 10, ch-4009 Basel, Switzerland: Karger.

[23] Food and Agricultural Organisation of the United Nations. Hunger and food insecurity. Available from: http://www.fao.org/hunger/en/#:~:text=A%20person%20is%20food%20insecure,of%20resources%20to%20obtain%20food. Accessed Oct 2020.

[24] Carter KN, Lanumata T, Kruse K, Gorton D (2010). What are the determinants of food insecurity in New Zealand and does this differ for males and females?. Aust N Z J Public Health.

[25] Rose D (1999). Economic determinants and dietary consequences of food insecurity in the United States. J Nutr.

[26] Gundersen C, Engelhard E, Hake M (2017). The determinants of food insecurity among food bank clients in the United States. J Consum Aff.

[27] Marmot M, Wilkinson R, editors. Social determinants of health. Oup Oxford; 2005 Oct 13.

[28] Kohler U, Karlson KB, Holm A (2011). Comparing coefficients of nested nonlinear probability models. Stand Genomic Sci.

[29] University of Essex, Institute for Social and Economic Research. (2021). Understanding society: Waves 1-11, 2009-2020 and Harmonised BHPS: Waves 1-18, 1991-2009. [data collection]. 14th Edition. UK Data Service. SN: 6614, 10.5255/UKDA-SN-6614-15.

[30] Office of National Statistics. Average household income, UK: financial year 2020. Available from: https://www.ons.gov.uk/peoplepopulationandcommunity/personalandhouseholdfinances/incomeandwealth/bulletins/householddisposableincomeandinequality/financialyear2020#:~:text=Main%20points,(ONS)%20Household%20Finances%20Survey. Accessed Feb 2022.

[31] Hawkes C, Squires CG (2021). A double-duty food systems stimulus package to build back better nutrition from COVID-19. Nature Food.

[32] Bae JH, Choi JH (2021). Gender disparities in childhood obesity and household food insecurity. Nutrition.

[33] Kaur J, Lamb MM, Ogden CL (2015). The association between food insecurity and obesity in children—The National Health and Nutrition Examination Survey. J Acad Nutr Diet.

[34] Pan L, Sherry B, Njai R, Blanck HM (2012). Food insecurity is associated with obesity among US adults in 12 states. J Acad Nutr Diet.

[35] Morales ME, Berkowitz SA (2016). The relationship between food insecurity, dietary patterns, and obesity. Current Nutr Rep.

[36] Department of Health and Social Care . Tackling obesity: empowering adults and children to live healthier lives. Available from: https://www.gov.uk/government/publications/tackling-obesity-government-strategy/tackling-obesity-empowering-adults-and-children-to-live-healthier-lives#what-next. Accessed Oct 2020.

[37] Milovanska-Farrington S. Job loss and food insecurity during the COVID-19 pandemic. 2021. Available from: http://ftp.iza.org/dp14273.pdf/. Accessed Apr 2021.

[38] Office of Budget Responsibility. The economy forecast. 2021 Available from: https://obr.uk/forecasts-in-depth/the-economy-forecast/. Accessed May 2021.

[39] OECD. Paid sick leave to protect income, health and jobs through the COVID-19 crisis. Available from: https://www.oecd.org/coronavirus/policy-responses/paid-sick-leave-to-protect-income-health-and-jobs-through-the-covid-19-crisis-a9e1a154/. Accessed Apr 2021.

[40] Reed, S. & Palmer, B. (2021) To solitude: learning from other countries on how to improve compliance with self-isolation, Nuffield Trust. Available from: www.nuffieldtrust.org.uk/news-item/to-solitude-learning-from-other-countries-on-how-to-improve-compliance-with-self-isolation-1. Accessed Apr 2021.

[41] Patel J, Fernandes G, Sridhar D (2021). How can we improve self-isolation and quarantine for covid-19?. BMJ.

